# Providing HIV‐assisted partner services to partners of partners in western Kenya: an implementation science study

**DOI:** 10.1002/jia2.26280

**Published:** 2024-07-05

**Authors:** Monisha Sharma, Barbara Wanjiku Mambo, Hanley Kingston, George Otieno, Sarah Masyuko, Harison Lagat, David A. Katz, Beatrice Wamuti, Paul Macharia, Rose Bosire, Mary Mugambi, Edward Kariithi, Carey Farquhar

**Affiliations:** ^1^ Department of Global Health University of Washington Seattle Washington USA; ^2^ Ministry of Health Nairobi Kenya; ^3^ Institute of Public Health Genetics University of Washington Seattle WA USA; ^4^ PATH‐Kenya Kisumu Kenya; ^5^ School of Nursing University of Washington Seattle Washington USA; ^6^ School of Computing and Engineering Sciences Strathmore University Nairobi Kenya; ^7^ Centre for Public Health Research Kenya Medical Research Institute (KEMRI) Nairobi Kenya; ^8^ Department of Epidemiology University of Washington Seattle Washington USA; ^9^ Department of Medicine University of Washington Seattle Washington USA

**Keywords:** HIV testing, Kenya, assisted partner services, partner notification, sexual partners, Africa

## Abstract

**Introduction:**

Assisted partner services (APS), or exposure notification and HIV testing for sexual partners of persons diagnosed HIV positive (index clients), is recommended by the World Health Organization. Most APS literature focuses on outcomes among index clients and their partners. There is little data on the benefits of providing APS to partners of partners diagnosed with HIV.

**Methods:**

We utilized data from a large‐scale APS implementation project across 31 facilities in western Kenya from 2018 to 2022. Females testing HIV positive at facilities were offered APS; those who consented provided contact information for all male sexual partners in the last 3 years. Male partners were notified of their potential HIV exposure and offered HIV testing services (HTS). Males newly testing positive were also offered APS and asked to provide contact information for their female partners in the last 3 years. Female partners of male partners (FPPs) were provided exposure notification and HTS. All participants with HIV were followed up at 12 months post‐enrolment to assess linkage‐to antiretroviral treatment (ART) and viral suppression. We compared HIV positivity, demographics and linkage outcomes among female index clients and FPPs.

**Results:**

Overall, 5708 FPPs were elicited from male partners, of whom 4951 received HTS through APS (87% coverage); 291 FPPs newly tested HIV positive (6% yield), an additional 1743 (35.2%) reported a prior HIV diagnosis, of whom 99% were on ART at baseline. At 12 months follow‐up, most FPPs were taking ART (92%) with very few adverse events: <1% reported intimate partner violence or reported relationship dissolution. FPPs were more likely than female index clients to report HIV risk behaviours including no condom use at last sex (45% vs. 30%) and multiple partners (38% vs. 19%).

**Conclusions:**

Providing HIV testing via APS to FPP is a safe and effective strategy to identify newly diagnosed females and achieve high linkage and retention to ART and can be an efficient means of identifying HIV cases in the era of declining HIV incidence. The high proportion of FPPs reporting HIV risk behaviours suggests APS may help interrupt community HIV transmission via increased knowledge of HIV status and linkage to treatment.

## 1 INTRODUCTION

Despite significant strides in combating the HIV epidemic, progress towards the UNAIDS 95‐95‐95 targets has fallen short of projections and sub‐Saharan Africa (SSA) continues to have the highest global HIV burden [1]. HIV testing is the first crucial step to accessing lifesaving antiretroviral treatment (ART). Timely diagnosis can improve individual clinical outcomes and prevent onward transmission to partners [2]. However, approximately 14% of individuals with HIV globally are unaware of their status. In SSA, individuals with HIV are generally diagnosed through facility‐based HIV testing; however, testing rates have been insufficient to curb the epidemic [3]. With reductions in HIV funding and increased emphasis on high‐yield testing strategies, countries have begun reducing the volume of routine facility‐based HIV testing [4]. Innovative testing strategies are needed to efficiently target those at high risk of undiagnosed HIV.

Assisted partner services (APS), which entails exposure notification and HIV testing for sexual partners of persons diagnosed with HIV (index clients), is a targeted strategy which can address the gap in HIV testing coverage. Types of APS include (1) provider notification: providers contact partners to offer testing; (2) contract referral: index clients are given a certain period of time to notify partners, after which providers conduct notification and HIV testing; and (3) dual referral: both providers and index clients coordinate to notify partners. Several clinical trials and implementation projects have demonstrated the safety and effectiveness of APS in reaching HIV‐exposed partners with HIV testing and linkage to treatment, with low risk of adverse events among index clients and named partners [3, 5–9]. Additionally, APS can be a high‐yield strategy to identify new cases of HIV (12–23% positivity found in Kenya), although performance varies outside of trial settings [6, 10]. The World Health Organization (WHO) recommended offering APS as part of routine HIV services in 2016 [11]. Our team's APS scale‐up project, which integrated APS into 31 government facilities in western Kenya, found high uptake of APS among female index clients (90%) and their male partners (82%). At 12 months follow‐up, approximately 90% of female index clients and male partners with HIV were taking ART [10]. Despite substantial data on index clients and partner outcomes, little is known about the benefits of providing APS to partners of partners diagnosed with HIV, which is routinely conducted in APS programmes although it requires additional effort and resources. Partners of partners reached with HIV testing via APS may differ in demographics, risk behaviour and linkage to care. Understanding heterogeneity in APS performance across subgroups can inform guidelines to optimize programmes [12].

Within the APS scale‐up study in western Kenya [10], we assessed the real‐world performance of providing APS to female partners of male partners (FPPs) newly diagnosed with HIV. We assess demographics, APS uptake, positivity, linkage to ART, viral suppression and adverse events among FPPs compared to those of female index clients identified via facility testing.

## 2 METHODS

### 2.1 Study design

We utilized data from the APS Scale‐up Project, a single‐arm hybrid type II implementation science study [13] conducted between May 2018 and May 2022 in two counties in western Kenya (Homa Bay and Kisumu). This project was led by researchers from the University of Washington, PATH (a non‐governmental organization) and the Kenya Ministry of Health (MOH). The protocol and implementation strategies have been previously published [10, 14]. Briefly, we sought to implement and evaluate APS (intervention) when integrated into health facilities and conducted by government healthcare workers as part of their regular clinic duties (implementation strategy). Our hypothesis was that offering APS to FPPs would result in high HIV testing uptake, high linkage to ART among those testing positive and low rates of adverse events. Our implementing partner (PATH) provided training, mentorship and ongoing quality assurance to clinic staff via a team of eight health advisors with expertise in APS provision. The health advisors provided initial and ongoing staff trainings on topics including counselling, partner elicitation and tracing. Health advisor engagement was primarily provided during the first year of implementation and incrementally reduced over subsequent years. PATH also provided resources for phone and in‐person tracing of partners.

### 2.2 Study procedures

Although APS was offered to both males and females newly diagnosed with HIV (index clients), we only collected programme data for female index clients, their male partners and FPPs. HIV testing services (HTS) counsellors offered APS to eligible individuals at the time of HIV diagnosis. Females were eligible if they were ≥18 years of age (or emancipated minor ≥15 years), at low risk of intimate partner violence (IPV), not pregnant, newly diagnosed with HIV, not on ART and reported ≥1 sexual partner within the last 3 years. Prior to receiving APS, females were informed that participation was voluntary and refusal would not impact their clinical care. Consenting females were asked to provide names and contact information for all partners in the last 3 years. HTS counsellors contacted male partners via phone or in‐person to notify them of their potential exposure and offer HIV testing. Partners were told that they may have been exposed to HIV but were not provided details about the exposure nor identifying information about the index client. Male partners were eligible for testing via APS if they were ≥18 years of age (or emancipated minor ≥15 years). Males who were diagnosed with HIV were also offered APS and asked to provide contact information for their female partners. Female partners elicited by males who were the original index client who named the male were not contacted and re‐enrolled. HTS counsellors provided exposure notification and HIV testing for non‐index female partners of consenting males. Female partners were eligible for HIV testing via APS if they were ≥18 years of age (or emancipated minor ≥15 years). Participants were not reimbursed for their participation in APS. The IPV assessment (which was only administered to female index clients to reduce risks of adverse events associated with partner notification) is available in the Supporting information.

All partners diagnosed with HIV were encouraged to link to ART at a health facility. We followed up with enrolled female index clients and partners with HIV (both newly diagnosed or known to have HIV) at 6 weeks, 6 months and 12 months post enrolment via phone or in‐person to assess linkage to care, ART status, IPV and relationship dissolution. All reported instances of IPV and relationship dissolution were included, regardless of whether participants perceived them to be related to APS provision. ART status was assessed via a record review when possible. Viral load testing was conducted 12 months post enrolment. Participant demographics and HIV risk behaviours were collected at enrolment.

### 2.3 Study population

The present analysis includes 12 months of follow‐up data from female index clients enrolled in APS and female partners elicited from the male partners of female index clients enrolled from May 2018 to May 2022. Outcomes for male partners and female index clients enrolled from May 2018 to March 2020 have been previously published [10], but cumulative data on female index clients during the entire APS programme have not been reported and data from FPPs have not been previously published.

### 2.4 Data analysis

Outcomes included APS uptake, new HIV diagnosis and linkage to ART by 12 months for female index clients and female partners of partners. We also report adverse events, including IPV and relationship dissolution. We report continuous variables using median and interquartile ranges (IQR) and categorical variables using percentages; *p*‐values were calculated via *t*‐test (continuous variables) or chi‐square (categorical variables). Omnibus *p*‐values were calculated for variables with multiple categories. We calculated univariable and multivariable (adjusting for age group and enrolment county) prevalence ratios using negative binomial regression with robust standard errors to evaluate differences in characteristics between female index clients and FPPs. Negative binomial regression was chosen as it is a special case Poisson regression that relaxes the mean equals variance assumption [15]. Analyses were conducted using Stata Software V17 and R V4.2.3 [16, 17].

### 2.5 Ethical reviews

The study was approved by the Kenyatta National Hospital Ethics and Research Committee (ERC; P465/052017), the University of Washington Institutional Review Board (IRB; STUDY00002420) and the PATH Institutional Review Board.

## 3 RESULTS

### 3.1 Participant demographics

Across 31 facilities, 4951 non‐index female partners elicited from 4422 males received HIV testing through APS (Table 1). Among enrolled FPPs, 291 (6%) were newly diagnosed with HIV and an additional 1743 (35%) were known to have HIV. The median age of FPPs was 30 (IQR 26–33) years, most were married or living with one partner (72%), 7% were in polygamous marriages and 13% were never married. FPPs generally had low levels of education and income; most had some secondary school or lower (67%) and 81% reported household earnings of less than 10,000 KSh ($87 USD) per month. Overall, 72% of FPPs were employed, of whom 49% were self‐employed, and had a median of two sexual partners in the past 3 years (IQR 1–3). Most FPPs reported at least one HIV risk indicator in the past year (80%), the most common being inconsistent condom use (48%). The majority (95%) of FPPs reported having tested for HIV previously and 16% had used an HIV self‐test; most were tested in the community (70%). Very few FPPs reported transactional sex (0.5%), sex under the influence of drugs (0.7%) or injection drug use with needle‐sharing (<0.1%).

**Table 1:** Demographics, sexual behaviour and HIV testing outcomes of enrolled female partners of male partners receiving assisted partner services in western Kenya

	HIV negative (*n* = 2916, 58.9%)	Known to have HIV (*n* = 1743, 35.2%)	Newly diagnosed with HIV (*n* = 291, 6%)	Total (*N* = 4951)
Age (years)^a^	29 (26, 32)	30 (27, 35)	29 (24, 32.8)	30 (26, 33)
15–24 years	511 (17.5%)	188 (10.8%)	75 (25.8%)	774 (15.6%)
≥25 years	2405 (82.5%)	1554 (89.2%)	216 (74.2%)	4175 (84.4%)
Marital status				
Married monogamous/cohabitating	2068 (70.9%)	1300 (74.6%)	176 (60.5%)	3544 (71.6%)
Single/never married	458 (15.7%)	138 (7.9%)	61 (21.0%)	657 (13.3%)
Married polygamous	186 (6.4%)	140 (8.0%)	15 (5.2%)	341 (6.9%)
Divorced/separated	131 (4.5%)	75 (4.3%)	21 (7.2%)	227 (4.6%)
Widowed	73 (2.5%)	90 (5.2%)	18 (6.2%)	181 (3.7%)
Highest education completed				
Did not complete primary school	413 (14.2%)	229 (13.1%)	51 (17.5%)	693 (14.0%)
Completed primary school	694 (23.8%)	476 (27.3%)	63 (21.6%)	1233 (24.9%)
Some secondary school	733 (25.1%)	544 (31.2%)	90 (30.9%)	1367 (27.6%)
Completed secondary school	755 (25.9%)	366 (21.0%)	62 (21.3%)	1183 (23.9%)
Post‐secondary school	321 (11.0%)	128 (7.3%)	25 (8.6%)	474 (9.6%)
Primary occupation				
Formally employed	746 (25.8%)	335 (19.4%)	74 (25.9%)	1155 (23.5%)
Self‐employed	1309 (45.2%)	955 (55.3%)	118 (41.3%)	2382 (48.5%)
Unemployed	146 (5.0%)	61 (3.5%)	15 (5.2%)	222 (4.5%)
Student	146 (5.0%)	38 (2.2%)	16 (5.6%)	200 (4.1%)
Spouse‐supported	549 (19.0%)	337 (19.5%)	63 (22.0%)	949 (19.3%)
Monthly household income				
0–10,000 KSh	2361 (81.0%)	1399 (80.3%)	228 (78.4%)	3988 (80.6%)
>10,000	555 (19.0%)	344 (19.7%)	63 (21.6%)	962 (19.4%)
Key population^b^				
Female sex workers	2 (<0.1%)	0 (0%)	0 (0%)	2 (<0.1%)
Fisherfolk^c^	3 (0.1%)	8 (0.5%)	0 (0%)	11 (0.2%)
Adolescent girls and young women (age 15–24)	178 (6.1%)	40 (2.3%)	26 (8.9%)	244 (4.9%)
Have experienced IPV		1 (0.1%)	0 (0%)	1 (<0.1%)
County				
Kisumu	669 (22.9%)	340 (19.5%)	69 (23.7%)	1078 (21.8%)
Homabay	2247 (77.1%)	1403 (80.5%)	222 (76.3%)	3872 (78.2%)
Risk indicators last 12 months^d^				
Inconsistent condom use	1383 (47.4%)	–	145 (49.8%)	1528 (47.6%)
No condom in last sex	1309 (44.9%)	–	132 (45.4%)	1441 (44.9%)
Ever used PrEP	170 (5.8%)	–	21 (7.2%)	191 (6.0%)
Recurrent PEP use	152 (5.2%)	–	20 (6.9%)	172 (5.4%)
Recent STI	168 (5.8%)	–	25 (8.6%)	193 (6.0%)
Multiple sexual partners	935 (32.1%)	–	111 (38.1%)	1046 (32.6%)
HIV‐positive sexual partner	185 (6.3%)	–	11 (3.8%)	196 (6.1%)
High HIV risk sexual partners	29 (1.0%)	–	4 (1.4%)	33 (1.0%)
Transactional sex	15 (0.5%)	–	2 (0.7%)	17 (0.5%)
Sex under influence of drugs	18 (0.6%)	–	6 (2.1%)	24 (0.7%)
IDU sharing needle	1 (<0.1%)	–	0 (0.0%)	1 (<0.1%)
Inconsistent condom use/no condom at last sex AND multiple sex partners	836 (28.7%)		102 (35.1%)	938 (29.3%)
Any risk indicators in the last 12 months^d^				
None	579 (19.9%)	–	75 (25.8%)	655 (20.4%)
At least one	2337 (80.1%)	–	216 (74.2%)	2553 (79.6%)
Sexual partners in the past 3 years^a^	–	1 (1,1.2)	1 (1,1)	1 (1,1)
≤1	–	1251 (75.0%)	224 (77.8%)	1481 (75.4%)
>1	–	417 (25.0%)	64 (22.2%)	484 (24.6%)
Previously tested for HIV	2711 (93.0%)	1711 (98.2%)	270 (92.8%)	4692 (94.8%)
Self‐tested for HIV in the last 12 months	696 (23.9%)	52 (3.0%)	47 (16.2%)	795 (16.1%)
Tested as a couple during APS	20 (0.7%)	–	4 (1.4%)	24 (0.7%)
HIV testing location for APS^e^		–		
Facility based	1448 (49.7%)	–	63 (21.6%)	1511 (47.1%)
Community based	1468 (50.3%)	–	228 (78.4%)	1696 (52.8%)

Abbreviations: aPR, adjusted prevalence ratio; APS, assisted partner notification services; FPP, female partners of male partners; IDU, injection drug use; IPV, intimate partner violence; Ksh, Kenyan Schillings; PEP, post‐exposure prophylaxis; PrEP, pre‐exposure prophylaxis; PR, prevalence ratio; STI, sexually transmitted infection.

^a^ Median, interquartile range (IQR).

^b^ No participant enrolled from the following key populations: transgender persons or people who inject drugs.

^c^ People who catch or sell fish for a living.

^d^ Question not asked for those who are known to have HIV.

^e^ All HIV tests done in the community were linked to a facility, and verification of the test results was completed at the facility's comprehensive care clinic before enrolment to care and treatment.

Compared to FPPs who tested HIV negative, FPPs newly diagnosed with HIV were more likely to be widowed (6.2% vs. 2.5%) or divorced/separated (7.2% vs. 4.5%), less likely to be married monogamously (60.5% vs. 71%), despite similar age demographics (omnibus *p*‐value<0.05, Table S1). Additionally, newly diagnosed FPPs were more likely be HIV tested via APS in the community compared to the facility (78% vs. 50%) and less likely to report one or more risk indicators for HIV (74% vs. 80%) than those who tested negative (*p*<0.05 for both comparisons). The two groups were similar in terms of history of HIV testing and income and had similar rates of enrolment by county.

Table 2 displays the characteristics of newly diagnosed FPPs compared to female index clients. Most (76%) newly diagnosed FPPs were recruited from Homa Bay county (compared to 46% of index clients), and assessment of differences between the two groups were adjusted for county and age group. Compared to female index clients, FPPs newly diagnosed with HIV were more likely to have higher education levels (aPR: 1.40, 95% CI: 1.11–1.78) and higher income (aPR: 1.48, 95% CI: 1.10–1.95) and were less likely to be unemployed (aPR: 0.30, 95% CI: 0.17–0.51) or self‐employed (aPR: 0.58, 95% CI: 0.43–0.78). Newly diagnosed FPPs were also more likely to report one or more HIV‐related risk indicators (aPR: 2.12, 95% CI: 1.64–2.78). In terms of HIV testing history, FPPs were more likely to have previously tested for HIV and used an HIV self‐test, but less likely to have tested as a couple through APS.

**Table 2:** Characteristics of newly HIV‐diagnosed female partners of male partners compared to female index clients receiving assisted partner services

	Newly diagnosed FPPs *N* = 291	Female index clients *N* = 2534	Newly diagnosed FPP versus female index clients	
			PR (95% CI)	aPR (95% CI) adjusted for age and county
Age (years)^a^	29 (24, 32.8)	28 (23–34)		
15–24 years	75 (25.8%)	822 (32.5%)	Ref	Ref
≥25 years	216 (74.2%)	1710 (67.5%)	**1.34 (1.04**–**1.75)**	**1.34 (1.03**–**1.76)**
Marital status				
Married monogamous/cohabitating	176 (60.5%)	1533 (60.5%)	Ref	Ref
Single/never married	61 (21.0%)	471 (18.6%)	1.11 (0.83–1.48)	**1.63 (1.18**–**2.22)**
Married polygamous	15 (5.2%)	157 (6.2%)	0.85 (0.48–1.38)	0.81 (0.46–1.33)
Divorced/separated	21 (7.2%)	185 (7.3%)	0.99 (0.61–1.52)	1.02 (0.63–1.56)
Widowed	18 (6.2%)	188 (7.4%)	0.85 (0.5–1.34)	0.64 (0.38–1.02)
Highest education completed				
Primary or below			Ref	Ref
Did not complete primary school	51 (17.5%)	628 (24.8%)	–	–
Completed primary school	63 (21.6%)	614 (24.2%)	–	–
More than primary			**1.43 (1.13**–**1.82)**	**1.40 (1.11**–**1.78)**
Some secondary school	90 (30.9%)	578 (22.8%)		
Completed secondary school	62 (21.3%)	490 (19.3%)	–	–
Post‐secondary school	25 (8.6%)	224 (8.8%)	–	–
Occupation				
Formally employed	74 (25.9%)	380 (15.3%)	Ref	Ref
Self‐employed	118 (41.3%)	1112 (44.7%)	**0.59 (0.44**–**0.79)**	**0.58 (0.43**–**0.78)**
Unemployed	15 (5.2%)	404 (16.2%)	**0.22 (0.12**–**0.37)**	**0.30 (0.17**–**0.51)**
Student	16 (5.6%)	121 (4.9%)	0.72 (0.40–1.20)	0.98 (0.53–1.69)
Spouse‐supported	63 (22.0%)	472 (19.0%)	0.72 (0.52–1.01)	**0.65 (0.46**–**0.90)**
Monthly household income				
0–10,000 KSh	228 (78.4%)	2068 (81.6%)	Ref	Ref
>10,000	63 (21.6%)	466 (18.4%)	1.20 (0.90–1.57)	**1.48 (1.10**–**1.95)**
Key population^b^				
Female sex workers	0 (0%)	5 (0.2%)	–	–
Fisherfolk	0 (0%)	14 (0.6%)	–	–
Adolescent girls and young women (age 15–24)	26 (8.9%)	310 (12.2%)	0.73 (0.47, 1.07)	0.99 (0.60–1.57)
Have experienced IPV	0 (0%)	40 (1.6%)	–	–
County				
Kisumu	69 (23.7%)	1371 (54.1%)	Ref	Ref
Homabay	222 (76.3%)	1163 (45.9%)	**3.35 (2.57**–**4.41)**	**3.34 (2.56**–**4.41)**
HIV risk indicators last 12 months^c^				
Inconsistent condom use	145 (49.8%)	865 (34.1%)	**1.78 (1.42**–**2.25)**	**1.63 (1.29**–**2.05)**
No condom at last sex	132 (45.4%)	753 (29.7%)	**1.82 (1.44**–**2.29)**	**1.54 (1.22**–**1.94)**
Ever used PrEP	21 (7.2%)	50 (2.0%)	**9.48 (5.89**–**14.39)**	**6.55 (4.06**–**9.99)**
Recurrent PEP use	20 (6.9%)	3 (0.1%)	**8.99 (5.53**–**13.78)**	**6.29 (3.85**–**9.67)**
Recent STI	25 (8.6%)	50 (2.0%)	**3.45 (2.23**–**5.08)**	**2.48 (1.60**–**3.67)**
Multiple sexual partners	111 (38.1%)	482 (19.0%)	**2.32 (1.82**–**2.93)**	**1.93 (1.52**–**2.45)**
HIV‐positive sexual partner	11 (3.8%)	51 (2.0%)	1.75 (0.90–3.04)	1.76 (0.91–3.06)
High HIV risk sexual partners	4 (1.4%)	113 (4.5%)	–	–
Transactional sex	2 (0.7%)	12 (0.5%)	–	–
Sex under influence of drugs	6 (2.1%)	24 (0.9%)	–	–
IDU sharing needle	0 (0.0%)	1 (<0.1%)	–	–
Inconsistent condom use/no condom at last sex AND multiple sex partners	102 (35.1%)	393 (15.5%)	–	–
Any risk indicators last 12 months				
None	75 (25.8%)	1205 (47.6%)	Ref	Ref
At least one	216 (74.2%)	1332 (52.6%)	**2.39 (1.84**–**3.12)**	**2.12 (1.64**–**2.78)**
Sexual partners in the past 3 years^a^	1 (1,1)	3 (2–4)		
≤1	224 (77.8%)	360 (14.2%)	Ref	Ref
>1	64 (22.2%)	2174 (85.8%)	**0.07 (0.06**–**0.10)**	**0.07 (0.05**–**0.10)**
Previously tested for HIV	270 (92.8%)	2121 (83.7%)	**2.33 (1.54**–**3.75)**	**1.55 (1.01**–**2.51)**
Self‐tested for HIV in the last 12 months	47 (16.2%)	192 (7.6%)	**2.08 (1.51**–**2.82)**	**2.03 (1.47**–**2.74)**
Tested as a couple during APS	4 (1.4%)	103 (4.1%)	**0.35 (0.11**–**0.83)**	**0.35 (0.11**–**0.83)** ^d^
HIV testing location for APS^e^				
Facility based	63 (21.6%)	2097 (82.8%)	Ref	Ref
Non‐facility based	228 (78.4%)	437 (17.2%)	**11.76 (8.95**–**15.66)**	**12.64 (9.62**–**16.86)**

Abbreviations: aPR, adjusted prevalence ratio; APS, assisted partner notification services; FPP, female partners of male partners; IDU, injection drug use; IPV, intimate partner violence; Ksh, Kenyan Schillings; PEP, post‐exposure prophylaxis; PrEP, pre‐exposure prophylaxis; PR, prevalence ratio; STI, sexually transmitted infection; Ref, reference.

^a^ Values in bold are statistically significant at *p* = 0.05, median, interquartile range (IQR), PR not calculated for cells with *N*<5.

^b^ People who catch or sell fish for a living.

^c^ Question not asked for those who are known HIV positive.

^d^ No participant enrolled from the following key populations: bisexual/MSM, transgender persons or people who inject drugs.

^e^ All HIV tests done in the community were linked to a facility, and verification of the test results was completed at the facility's comprehensive care clinic before enrolment to care and treatment.

### 3.2 APS care cascade

APS uptake and linkage to ART was high among both female index clients and FPPs (Table 3). Overall, 5708 non‐index FPPs were elicited from male partners, of whom 4951 (87%) received HIV testing through APS and 291 (6% of those enrolled) were newly diagnosed with HIV. Among 1743 FPPs reporting a previous HIV diagnosis (known to have HIV), >99% self‐reported being on ART at baseline. At 12 months follow‐up, ART linkage was high among female index clients (90%), newly diagnosed FPPs (90%) and FPPs known to have HIV (92%); participants lost to follow‐up made up the majority of those not confirmed to be on ART. Of individuals on ART at 12 months, viral load data were not available for 22% of female index clients, 51% of newly diagnosed FPPs and 11% of FPPs known to have HIV. Conservatively assuming a lack of viral suppression among those without viral load data on ART, an estimate of 73% of female index clients, 48% of newly diagnosed FPPs and 88% of FPPs known to have HIV on ART at 12 months were virally suppressed, with viral suppression rates being statistically different across groups (omnibus *p*‐value<0.05). Among those with viral load results available, 94% of female index clients, 98.4% of newly diagnosed FPPs and 99.2% of FPPs known to have HIV were virally suppressed. Reported IPV was low at 12 months (1.5% of females index clients and 0.5% of FPPs) as was relationship dissolution (2.6% of female index clients and 0.2 of FPPs).

**Table 3:** APS care cascade and outcomes among female partners of male partners and female index clients receiving assisted partner services

	Female partners of male partners		Female index clients	
APS cascade	N	%	N	%
Elicited (FPP) or tested (female index clients)	5708		42,579	–
Traced and enrolled (/elicited)	4951	*(87* *%)*	–	
HIV positive (/enrolled [FPP]/tested [female index clients])	2031	*(41%)*	2778	*(7%)*
Enrolled in APS (/HIV positive)	–	2534	*(91%)*	
FPPs with a prior HIV diagnosis (/enrolled)	1742	*(35%)*	–	
On ART at APS enrolment (/prior HIV diagnosis)	1736	*(99.7%)*	–	
Newly diagnosed HIV positive (/enrolled)	289	*(6%)*	–	
**12‐month outcomes**				
Attended 12‐month follow up (/enrolled)	1868	*(92%)*	2319	*(92%)*
On ART (/enrolled)	1862	*(92%)*	2286	*(90%)*
VL results available (/enrolled)	1554	*(77%)*	1788	*(71%)*
Virally suppressed (/VL results available)	1541	*(82%)*	1673	*(94%)*
Relationship ended since APS enrolment (/attended 12 months follow up)^a^	4	*(0.2%)*	69	*(2.6%)*
Experienced intimate partner violence since APS enrolment (/attended 12 months follow up)	10	*(0.5%)*	34	*(1.5%)*
FPPs with a prior HIV diagnosis (*N* = 1742)	1607	*(92%)*	–	
On ART (/enrolled)	1603	*(92%)*	–	
Viral load results available (/enrolled)	1427	*(82%)*	–	
Virally suppressed (/VL results available)	1415	*(99%)*	–	
Newly diagnosed HIV‐positive FPPs (*N* = 289)	261	(90%)	–	
On ART (/enrolled)	259	*(90%)*	–	
Viral load results available (/enrolled)	127	*(44%)*	–	
Virally suppressed (/VL results available)	125	*(98%)*	–	

Italic values are the percentages of the number reported divided by the denominator listed in each row.

Abbreviations: APS, Assisted partner service; ART, antiretroviral treatment; FPP, female partners of male partners; VL, viral load.

^a^ Participant can report more than one instance of IPV or relationship dissolution.

## 4 DISCUSSION

We found that providing APS to FPPs within a large‐scale implementation project in western Kenya was a safe and effective strategy to reach potentially HIV‐exposed females with testing and treatment. The high uptake of HIV testing via APS among FPPs (87%), high retention on ART at 12 months (>90%) and low rates of adverse events suggest that APS is an effective strategy to reach HIV treatment targets. Our findings are similar to the uptake and linkage rates observed among male partners in our study and those of other APS demonstration projects in SSA [5, 8, 10]. As donor funding declines in SSA, targeted HIV testing strategies to identify undiagnosed persons with HIV are increasingly crucial. The yield of newly diagnosed FPPs was lower than observed in male partners in our APS programme (6% vs. 12%), despite higher population HIV prevalence in females compared to males; overall HIV positivity was similar in the two groups but a greater proportion of FPPs was known to have HIV and already on ART. This is consistent with studies showing that females in SSA are more likely to access timely HIV testing and linkage to treatment compared to males [18]. Further, some FPPs named by male clients were the original index client so were not included in HIV positivity estimates. However, we enrolled and followed up all FPPs with HIV, whereas in APS programmes run by governments or NGOs, services would likely not be offered to those who know their HIV status. Removing females with known HIV from our denominator results in a yield of 9%, which is higher than that found through facility‐based testing [19].

FPPs newly diagnosed with HIV tended to have higher income and education than newly diagnosed female index clients identified through facility‐based testing. Newly diagnosed FPPs were also more likely to report at least one risk indicator for HIV. Taken together, this suggests that APS may be reaching a different population of females with HIV than routine facility testing. Additionally, the high prevalence of risk indicators among FPPs unaware that they have HIV suggests that testing via APS may be efficient in preventing onward HIV transmission. In particular, 45% of newly diagnosed FPPs reported no condom use during last sex and 38% reported multiple sexual partners, indicating activity that may put partners at risk for HIV. Approximately 80% of FPPs newly diagnosed with HIV received community testing compared to 50% of those testing HIV negative, highlighting the importance of client‐centred testing strategies that overcome barriers to healthcare access to reach persons with undiagnosed HIV. Community‐based testing has also been shown to reach individuals with HIV earlier in their disease course (as determined by CD4 count) [3]; however, it is not possible to assess whether APS reached FPPs at an earlier stage compared to female index clients in this study. Future research may consider using HIV recency testing to target additional testing among sexual networks or inform HIV incidence estimates [20].

Interestingly, FPPs testing HIV negative had the highest rates of reported HIV risk indicators (compared to both newly diagnosed FPPs and index clients). This highlights the potential of leveraging APS to reach HIV‐negative females at substantial risk of HIV with prevention, particularly pre‐exposure prophylaxis (PrEP). Females in SSA are disproportionately impacted by HIV, accounting for 63% of all new HIV acquisitions and targeted prevention strategies are urgently needed to reach females at substantial risk [21]. Despite high effectiveness, PrEP uptake has been low among those at HIV risk. The WHO recommends a differentiated service delivery approach tailored to the needs of those vulnerable to HIV [22]. Prior use of both PrEP and post‐exposure prophylaxis was higher among FPPs compared to female index clients (6% vs. 2.0%) indicating the acceptability of biomedical prevention. Further studies are needed to evaluate the effectiveness of utilizing APS to increase PrEP coverage.

Consistent with previous studies, we find low rates of IPV and relationship dissolution among individuals at 12 months after APS [6, 8, 23], suggesting that APS is associated with a low risk of social harms. However, reported rates of IPV are low; this may be due to the sensitive nature of the questions, and responses may be subject to social desirability bias. Further, social harms were only assessed among females who were successfully followed up at 12 months post APS; rates may be higher among the 8% of female partners not followed up. Ongoing provider training on counselling strategies for IPV screening and continued surveillance is needed to monitor rates of adverse events as APS is scaled up. Additionally, rates of relationship dissolution were higher among female index clients than FPPs, possibly because HIV testing via APS may have less impact on individuals’ relationships than partner notification.

Viral suppression at 12 months was high among individuals with viral load results available. However, a substantial number of females were missing viral load results, likely due to the start of the COVID‐19 pandemic, during which viral load testing was largely halted in Kenya. In particular, newly diagnosed FPPS had the highest proportion of missing viral load data (with almost half missing). We find the highest viral suppression rates among FPPs known to have HIV, consistent with prior studies showing the lowest viral suppression in the first year of ART [24]. The high retention on ART (>90%) at 12 months is reassuring but more research to assess viral suppression as the considerable amount of missing data adds uncertainty to our results.

Our study has several limitations. First, the data are from an APS programme so the quality is less robust than that of a clinical trial. Further, our sample has low representation from key populations (e.g. female sex workers and persons who inject drugs) which limits generalizability to these groups. Additionally, we do not assess APS performance in same‐sex partnerships. We also excluded female index clients at high risk of IPV and those who were pregnant so we cannot assess APS performance in these groups. Future studies are needed to understand the characteristics of females excluded due to IPV risk. Additionally, pregnant women diagnosed with HIV were excluded since they are a vulnerable population and inadvertent status disclosure to their partner could result in relationship dissolution and financial insecurity for themselves and their children. However, prior studies have found that partner testing among pregnant women with HIV was not associated with IPV or other adverse events [25]. Further, although we report findings from a real‐world implementation programme, we invested considerable funds in capacity building, staff training and resources for conducting partner tracing, which has been previously described [10, 14]. Therefore, results likely reflect the upper bound of those achievable in an APS programme. Continued provider training is crucial to APS sustainability. Additionally, self‐reported HIV risk indicators and sexual behaviour may be subject to social desirability bias. For example, only 25% of FPPs newly diagnosed with HIV named more than one sexual partner in the past 3 years (compared with 86% of female index clients) despite a higher proportion of FPPs reporting multiple partners compared to female index clients (38% vs. 19%). Ongoing provider training on partner elicitation may be needed in APS programmes.

Strengths of this study include its design and implementation via a partnership across the Kenya Ministry of Health, PATH (a non‐governmental implementing partner) and academic researchers. APS was conducted by government‐employed healthcare workers who also provided general HIV services at the facilities, enabling a real‐world assessment of APS scale‐up outside of trial settings. Individuals were not reimbursed for APS participation so uptake and retention on ART is likely reflective of programmatic settings.

## 5 CONCLUSIONS

We find that utilizing APS programmes to reach FPPs is a safe and effective strategy to reach HIV‐exposed females with testing and linkage to care. In an era of declining HIV incidence and reductions in HIV funding, APS is a targeted strategy to further progress to global targets to reduce the HIV burden in SSA.

## COMPETING INTERESTS

The authors declare that they have no competing interests.

## AUTHORS’ CONTRIBUTIONS

CF, EK, MS and BWM conceived of the analysis. HL, GO, DAK, SM, RB, MM and BW conducted data collection and management. HK, MS and BWM conducted the analyses. MS wrote the first draft of the paper with support from BWM. All authors had full access to the data, critically revised the manuscript and had final responsibility for the decision to submit for publication.

## FUNDING

This study was funded by the U.S. National Institutes of Health (NIH) NIAID R01AI134130. MS received support from NIMH K01MH115789. SM was supported by the Fogarty International Center: D43 TW009580, D43 TW009783 and D43 TW010905. The funders had no role in the study design, data collection and analysis, decision to publish or preparation of the article.

## CME STATEMENT

This article is published as part of a supplement supported by unrestricted educational grant by ViiV Healthcare.

Credits Available for this Activity: American Medical Association (AMA Credit).

Washington University School of Medicine in St. Louis designates this enduring material for a maximum of 1 AMA PRA Category 1 Credit™. Physicians should claim only the credit commensurate with the extent of their participation in the activity.

## Supporting information


**Table S1**. Demographics, sexual behaviour, and HIV testing outcomes of enrolled female partners of male partners receiving assisted partner services in western Kenya
**Table S2**. Type of APS selected among female index clients for all partners named

## Data Availability

The APS Scale up Project protocol and de‐identified data are available upon request from the corresponding author.
