# Supplementary material for: Providing HIV‐assisted partner services to partners of partners in western Kenya: an implementation science study
Source: J Int AIDS Soc. 2024 Jul 5;27(Suppl 1):e26280. doi: 10.1002/jia2.26280 (PMC11224583; doi:10.1002/jia2.26280)
Supplement: Supplementary file 1 — Table S1. Demographics, sexual behaviour, and HIV testing outcomes of enrolled female partners of male partners receiving assisted partner services in western Kenya Table S2. Type of APS selected among female index clients for all partners named [file JIA2-27-e26280-s001.docx]

**SUPPLEMENTAL APPENDIX**

**Accompanying the manuscript**

**Providing HIV assisted partner services to partners of partners in Kenya: An implementation science study**

Table S1. Demographics, sexual behavior, and HIV testing outcomes of enrolled female partners of male partners receiving assisted partner services in western Kenya

|  | HIV negative  (N=2916, 58.9%) | Newly diagnosed with HIV (N=291, 6%) | P^α^  (HIV negative v. newly-diagnosed |
| --- | --- | --- | --- |
| Age (years) ^β^ | 29 (26, 32) | 29 (24, 32.8) | 0.71 |
| 15-24 years | 511 (17.5%) | 75 (25.8%) |  |
| ≥25 years | 2405 (82.5%) | 216 (74.2%) |  |
| Marital status |  |  | <0.001 |
| Married monogamous / cohabitating | 2068 (70.9%) | 176 (60.5%) |  |
| Single/never married | 458 (15.7%) | 61 (21.0%) |  |
| Married polygamous | 186 (6.4%) | 15 (5.2%) |  |
| Divorced/separated | 131 (4.5%) | 21 (7.2%) |  |
| Widowed | 73 (2.5%) | 18 (6.2%) |  |
| Highest Education Completed |  |  | 0.09 |
| Did not complete primary school | 229 (13.1%) | 51 (17.5%) |  |
| Completed primary school | 476 (27.3%) | 63 (21.6%) |  |
| Completed secondary school | 910 (52.2%) | 152 (52.2%) |  |
| Post-secondary school | 128 (7.3%) | 25 (8.6%) |  |
| Primary occupation |  |  | 0.67 |
| Formally employed | 746 (25.8%) | 74 (25.9%) |  |
| Self-employed | 1309 (45.2%) | 118 (41.3%) |  |
| Unemployed | 146 (5.0%) | 15 (5.2%) |  |
| Student | 146 (5.0%) | 16 (5.6%) |  |
| Spouse-supported | 549 (19.0%) | 63 (22.0%) |  |
| Monthly household income |  |  | 0.28 |
| 0 to 10,000 KSh | 2361 (81.0%) | 228 (78.4%) |  |
| >10,000 | 555 (19.0%) | 63 (21.6%) |  |
| Key population* |  |  | - |
| Female sex workers | 2 (<0.1%) | 0 (0%) |  |
| Fisherfolk^£^ | 3 (0.1%) | 0 (0%) |  |
| Adolescent girls and young women (age 15-24) | 178 (6.1%) | 26 (8.9%) | 0.06 |
| Have experienced IPV |  | 0 (0%) | - |
| County |  |  | 0.77 |
| Kisumu | 669 (22.9%) | 69 (23.7%) |  |
| Homabay | 2247 (77.1%) | 222 (76.3%) |  |
| Risk indicators last 12 months^§^ |  |  |  |
| Inconsistent condom use | 1383 (47.4%) | 145 (49.8%) |  |
| No condom in last sex | 1309 (44.9%) | 132 (45.4%) |  |
| Ever used PrEP | 170 (5.8%) | 21 (7.2%) |  |
| Recurrent PEP use | 152 (5.2%) | 20 (6.9%) |  |
| Recent STI | 168 (5.8%) | 25 (8.6%) |  |
| Multiple sexual partners | 935 (32.1%) | 111 (38.1%) |  |
| HIV positive sexual partner | 185 (6.3%) | 11 (3.8%) |  |
| High HIV risk sexual partners | 29 (1.0%) | 4 (1.4%) |  |
| Transactional sex | 15 (0.5%) | 2 (0.7%) |  |
| Sex under influence of drugs | 18 (0.6%) | 6 (2.1%) |  |
| IDU sharing needle | 1 (<0.1%) | 0 (0.0%) |  |
| Inconsistent condom use/no condom at  last sex AND multiple sex partners | 836 (28.7%) | 102 (35.1%) |  |
| Any risk indicators in the last 12 months^§^ |  |  | 0.015 |
| None | 579 (19.9%) | 75 (25.8%) |  |
| At least 1 | 2337 (80.1%) | 216 (74.2%) |  |
| Sexual partners in the past 3 years ^β^ | - | 1 (1,1) | 0.43 |
| ≤1 | - | 224 (77.8%) |  |
| >1 | - | 64 (22.2%) |  |
| Previously tested for HIV | 2711 (93.0%) | 270 (92.8%) | 0.91 |
| Self-tested for HIV in last 12 months | 696 (23.9%) | 47 (16.2%) | 0.003 |
| Tested as a couple during APS | 20 (0.7%) | 4 (1.4%) | 0.19 |
| HIV testing location for APS^†^ |  |  | <0.001 |
| Facility based | 1448 (49.7%) | 63 (21.6%) |  |
| Non-facility based | 1468 (50.3%) | 228 (78.4%) |  |

^α^calculated via t-test (continuous) or chi-square (categorical). In the case of multiple categories, p-value indicates whether at least one group is statistically significantly different across outcomes.

^β^Median, interquartile range (IQR)

*No participant enrolled from the following key populations: transgender persons, or people who inject drugs

^£^People who catch or sell fish for a living.

^§^Question not asked for those who are known HIV positive

^†^All HIV tests done in the community were linked to a facility, and verification of the test results was completed at the facility’s comprehensive care clinic before enrolment to care and treatment”

**APS enrollment survey: Intimate Partner Violence Assessment**

Have you ever been in a relationship with a person who has physically hurt you?

If yes, when did this occur?

Have you been in a relationship with a person who threatens, frightens, or insults you, or treats you badly?

If yes, when did this occur?

Have you been in a relationship with a person who forces you to participate in sexual activities that make you feel uncomfortable?

If yes, when did this occur?

Do you think any of these things could happen to you if you decide to receive assisted partner notification services?

Table S2. Type of APS selected among female index clients for all partners named*

| APS method chosen by index client | N |  |
| --- | --- | --- |
| Provider | 7827 | 92.3% |
| Contract | 74 | 0.9% |
| Dual | 164 | 1.9% |
| Client | 307 | 3.6% |
| Couples testing | 105 | 1.2% |
| Total partners named | 8483 |  |

*Clients could select a different APS referral type for each partner.
